# MFAP2 promotes HSCs activation through FBN1/TGF‐β/Smad3 pathway

**DOI:** 10.1111/jcmm.17884

**Published:** 2023-08-27

**Authors:** Yonghong Sun, Xingxing Chen, Lili Chen, Baixin Bao, Chunming Li, Yongning Zhou

**Affiliations:** ^1^ Department of Gastroenterology The First Hospital of Lanzhou University Lanzhou China; ^2^ Department of Pediatrics Gansu Province People's Hospital Lanzhou People's Republic of China; ^3^ The First School of Clinical Medicine Gansu University of Chinese Medicine Lanzhou People's Republic of China; ^4^ Department of Obstetrics Gansu Province People's Hospital Lanzhou People's Republic of China

**Keywords:** FBN1, HSCs, liver fibrosis, MFAP2, TGF‐β1

## Abstract

Liver fibrosis is a chronic inflammatory process characterized by the accumulation of extracellular matrix (ECM), which contributes to cirrhosis and hepatocellular carcinoma. Increasing evidence suggests that the activation of hepatic stellate cells (HSCs) under an inflammatory state leads to the secretion of collagens, which can cause cirrhosis. In this study, we analysed data from the Gene Expression Omnibus (GEO) databases to identify differentially expressed genes (DEGs) between quiescent and fibrotic HSCs. We found that Microfibril Associated Protein 2 (MFAP2) was elevated in carbon tetrachloride (CCl4)‐induced liver fibrosis and Transforming Growth Factor‐Beta 1 (TGF‐β1)‐activated HSCs. Knockdown of MFAP2 inhibited HSC proliferation and partially attenuated TGF‐β‐stimulated fibrogenesis markers. Bioinformatics analysis revealed that Fibrillin‐1 (FBN1) was correlated with MFAP2, and the expression of FBN1 was significantly upregulated after MFAP2 overexpression. Silencing MFAP2 partially attenuated the activation of HSCs by inhibiting HSC proliferation and decreasing collagen deposits. In vitro results showed that the inhibition of MFAP2 alleviated hepatic fibrosis by inhibiting the activation and inducing the apoptosis of active HSCs in a CCl4‐induced mouse model. In conclusion, our results suggest that MFAP2 is a potential target for the clinical treatment of liver fibrosis.

## 1 INTRODUCTION

Liver fibrosis is a challenging global health problem caused by a wide range of chronic liver impairment, such as viral infections, excessive alcohol drinking and drug toxins.^1^, ^2^ Liver fibrosis is characterized by the excessive deposition of extracellular matrix (ECM) during wound‐healing response period and associated with progression to advanced liver cirrhosis and hepatocellular carcinoma (HCC).^3^ However, no specific efficacious therapy for liver fibrosis due to substantial progress in understanding the underlying mechanisms.

Hepatic stellate cells (HSCs) are the primary mesenchymal cells and would transform from a quiescent state to an active state following liver injury.^4^, ^5^ Numerous studies have reported that the activation and proliferation of HSCs contributed to accumulation of non‐functioning ECM.^6^, ^7^ Recent data have indicated that targeting HSC activation could reverse advanced fibrosis by inducing activated HSC apoptosis and inhibiting ECM production.^6^, ^8^ Therefore, identifying proper genes could provide new insights to slow the progression of liver fibrosis.

Microfibril‐associated protein 2 (MFAP2), a protein component of ECM microfibrils, has been reported to involve in elastic fibre formation, vascular and skeletal integrity, and energy metabolism of adipose tissue.^9^, ^10^, ^11^ It was found that MFAP2 interacted with microfibrillin to regulate microfibril functioning by binding active forms of transforming growth factor‐β (TGF‐β) and bone morphogenetic proteins (BMPs).^12^, ^13^ An increase expression of MFAP2 was observed in several malignant tumour tissues, including gastric cancer, head and neck squamous cell carcinomas and papillary thyroid cancer.^14^, ^15^, ^16^ TGF‐β1 is a well‐known pro‐fibrogenic factor predominantly produced by hepatocytes and Kupffer cells and promoted HSCs activation in liver during chronic inflammation.^8^, ^17^, ^18^ Furthermore, MFAP2 was found to promote epithelial–mesenchymal transition of gastric cancer cells by activating TGF‐β/SMAD2/3 signalling pathway.^14^ However, no studies have reported the function of MFAP2 in liver fibrosis.

In the current paper, GEO datasets were selected for genome‐wide gene expression profile analysis and identified MFAP2 as one of the differentially expressed genes (DEGs) between quiescent HSCs and fibrotic HSCs. Then the role of MFAP2 in liver fibrosis both in vivo and in vitro.

## 2 MATERIALS AND METHODS

### 2.1 Identification of DEGs from GEO

RNA sequencing (RNA‐Seq) expression data from GSE120281, GSE151251 and GSE176042 were downloaded from the GEO database (http://www.ncbi.nlm.nih.gov/geo). The online analysis software easyGEO (https://tau.cmmt.ubc.ca/eVITTA/easyGEO/#) was used to analyse the DEGs between the quiescent HSCs and fibrotic HSCs according to a threshold of adjusted *p* < 0.05 and a |log 2FC| ≥2. A heatmap was created to show the identified DEGs.

### 2.2 Cell line and culture conditions

The immortalized human hepatic stellate cell line LX‐2 was obtained from Cell bank of Chinese Academy of Sciences (Shanghai, China) and cultured in Dulbecco's modified Eagle's medium (DMEM) (Sigma‐Aldrich, St. Louis, MO, United States) containing 10% fetal bovine serum (Sigma‐Aldrich) and 1% penicillin/streptomycin. The cells were incubated at 37°C in a humidified atmosphere supplied with 5% CO_2_. LX‐2 cells were activated with 5 ng/mL recombinant human TGF‐β1 (Protein Tech Group, Inc, Chicago, USA). To inhibit the TGF‐β1/Smad3 signalling pathway, LX‐2 cells were treated with 10 μM SB431542 (type I TGF‐β1 receptor inhibitor) (Selleckchem, USA) for 24 h after MFAP2 overexpression.

### 2.3 RNA extraction and quantitative real‐time PCR (RT‐qPCR)

Total RNA was extracted using TRIzol reagents (Invitrogen, CA, USA) according to the manufacturer's instructions. Then, 1 μg of RNA was then transcribed into complementary DNA (cDNA) using PrimeScript™ RT reagent Kit (Takara Biomedical Technology Co., Ltd, Dalian, China). Target genes were quantified by quantitative real‐time PCR with TB Green® Fast qPCR Mix (Takara Biomedical Technology Co., Ltd) using the ABI PRISM 7900HT Sequence Detection System (Applied Biosystems, Foster City, CA, USA). All primers were synthesized by Sangon Biotech (Shanghai, China) and their sequences were shown at Table S1. The 2^−ΔΔCt^ methodology was employed in gene expression analysis and GAPDH was used as the housekeeping gene for normalization.

### 2.4 Construction of plasmids, lentivirus production and transduction

The cDNA of human MFAP2 and FBN1 were cloned into pCDH‐CMV‐MCS‐EF1α‐Puro expression lentivector and pcDNA™3.1 (+), respectively. An empty vector served as a negative control. Small hairpin RNAs (shRNAs) against MFAP2 were synthesized by Sangon Biotech. The MFAP2 shRNA was cloned into pLKO.1‐TRC cloning vector to produce plasmids termed shMFAP2#1 and shMFAP2#2, a non‐targeting shRNA served as a negative control.

For MFAP2 overexpression and knockout experiments, lentiviruses were generated by transfecting MFAP2, or shMFAP2#1 and shMFAP2#2 with packaging plasmids using FuGENE® 6 Transfection Reagent (Promega, Madison, WI, USA) in 293 T cells. Lentiviruses were harvested 72 h after transfection and filtered with a 0.45‐μm filter (Millipore, MA, USA). Cells were infected with lentiviruses with 8 μg/mL polybrene (Sigma‐Aldrich®, St. Louis, MI, USA) for 48 h and then selected by adding 2 μg/mL puromycin (Sigma‐Aldrich®).

### 2.5 Cell proliferation and apoptosis assay

Cells of each group were plated in 96‐well plates at a density of 2 × 10^3^ cells per well, 10 μL of the CCK‐8 reagent (Dojindo Molecular Technologies Inc., Japan) was added into each well and incubated at 37°C for 2 h. Then the absorbance value (optical density) was measured at 450 nm using a SPECTRAmax microplate spectrophotometer (Molecular Devices, Sunnyvale, CA, USA).

Cell apoptosis was assessed by flow cytometry assay using FITC Annexin V Apoptosis Detection Kit I (BD Biosciences, San Jose, CA, USA) according to the manufacturer's instructions. In brief, cells were harvested and stained with annexin V‐FITC and propidium iodide (PI). The apoptotic cells were measured using a BD FACSCalibur flow cytometer (BD Biosciences).

### 2.6 Haematoxylin–eosin and Masson staining

Fresh mouse liver tissues were fixed in 10% neutral buffer formalin (Sangon Biotech) for 24 h and embedded in paraffin. Then the liver sections were cut into a thickness of 5‐μm slices. Haematoxylin–eosin and Masson were performed to assess the liver pathology.

### 2.7 Biochemical analysis

At the end of the treatment, the mouse blood was collected from the eye socket and removed remaining cellular debris by centrifugating. Serum alanine aminotransferase (ALT) and aspartate aminotransferase (AST) were detected using the Automated Biochemical Analyser (AU‐680, Beckman, Germany).

### 2.8 Immunofluorescence staining

Cells were seeded on coverslip for 24 h, then cells were fixed in 10% neutral buffer formalin, permeabilized in 0.5% Triton X‐100 for 20 min. After blocking with 1% bovine serum albumin at room temperature. The slides were incubated with the primary antibody for double immunofluorescence staining overnight at 4°C. The primary antibodies used were as follows: rabbit anti‐α‐SMA (55135‐1‐AP, Proteintech, China) and rabbit anti‐COL1A1 (E6A8E) (#39952, Cell Signalling Technology, Boston, MA, USA). Subsequently, a CY3 or FITC‐conjugated secondary antibody was incubated at room temperature in the dark. Nuclei were stained with DAPI for 5 min under dark conditions. Images were captured using a confocal scanning microscope (Olympus).

### 2.9 Immunohistochemical staining

Immunohistochemical staining was performed as previously described.^19^

The mouse liver tissue sections were deparaffinized, hydrated, subjected to antigen retrieval and incubated with 0.3% hydrogen peroxide (H_2_O_2_) to block endogenous peroxidase activity. Then, the sections were incubated with primary antibodies against MFAP2 (PA5‐52425, Invitrogen, CA, USA), FBN 1 (ab53076, Abcam, Cambridge, England, UK), α‐SMA and COL1A1 overnight at 4°C. Finally, the sections were visualized with 0.05% 3,3‐diamino‐benzidine tetrachloride (DAB) and the sections were photographed under a light microscope (Nikon E400, Chiyoda, Tokyo, Japan).

### 2.10 Western blot

Cells of each group were harvested and lysed by RIPA lysis buffer (Cell Signalling Technology) containing phosphatase and protease inhibitors (Sigma‐Aldrich). Equal amounts (40 μg) of proteins were separated by 10% SDS‐PAGE and then transferred to polyvinylidene fluoride (PVDF) membranes (Bio‐Rad, Hercules, CA, USA). After blocking with 5% nonfat dry milk, the membranes were incubated with primary antibody against MFAP2, FBN1, Phospho‐Smad3 (Ser423/425) (C25A9) (#9520, Cell Signalling Technology), α‐SMA, COL1A1 and COL3A1 (#30565, Cell Signalling Technology) overnight at 4°C. The blots were visualized with enhanced chemiluminescence (ECL, Millipore, Boston, MA, USA) using a ChemiDoc Imaging System (BioRad Laboratories Inc.).

### 2.11 Experimental animals

C57BL/6J (8 weeks old) male mice were purchased from Department of Laboratory Animal of Shanghai ZY Inc (Shanghai, China). The mice were feed in s specific pathogen free environment (SPF). All animal experimental protocols were conducted in accordance with the Institutional Animal Care and Use Committee of SHZY (IACUC) Guide for Care and Use of Laboratory Animals (NO. SHZY‐202201253).

Mice were randomly categorized into five groups: Control group (normal saline), model group (CCl_4_ treatment, 10% CCl_4_, 1 mL/kg, twice a week for 8 weeks), SCR group (CCl_4_ treatment + lentivirus‐mediated negative control), sh‐MFAP2#1 (CCl_4_ treatment + lentivirus‐mediated short hairpin RNA targeting MFAP2) and sh‐MFAP2#2 (CCl_4_ treatment + lentivirus‐mediated short hairpin RNA targeting MFAP2). Meanwhile, during the induction period of chronic liver fibrosis, mice were injected with 1 × 10^8^ TU lentivirus via tail vein of once per week after 4 weeks CCl_4_ treatment.

### 2.12 Statistics analysis

Data are presented as the mean ± SD of at least three independent experiments. Two‐tailed unpaired Student's *t*‐test was used to analysed *p* value between two groups and a one‐way anova was used to analysed *p* value for multi‐group comparisons. All statistical analyses were performed using GraphPad Prism 5.0 program (GraphPad Software Inc.; San Diego, CA, USA). *p* < 0.05 was thought as significant statistically.

## 3 RESULTS

### 3.1 MFAP2 was upregulated in activated HSCs and liver fibrosis

**FIGURE 1:**
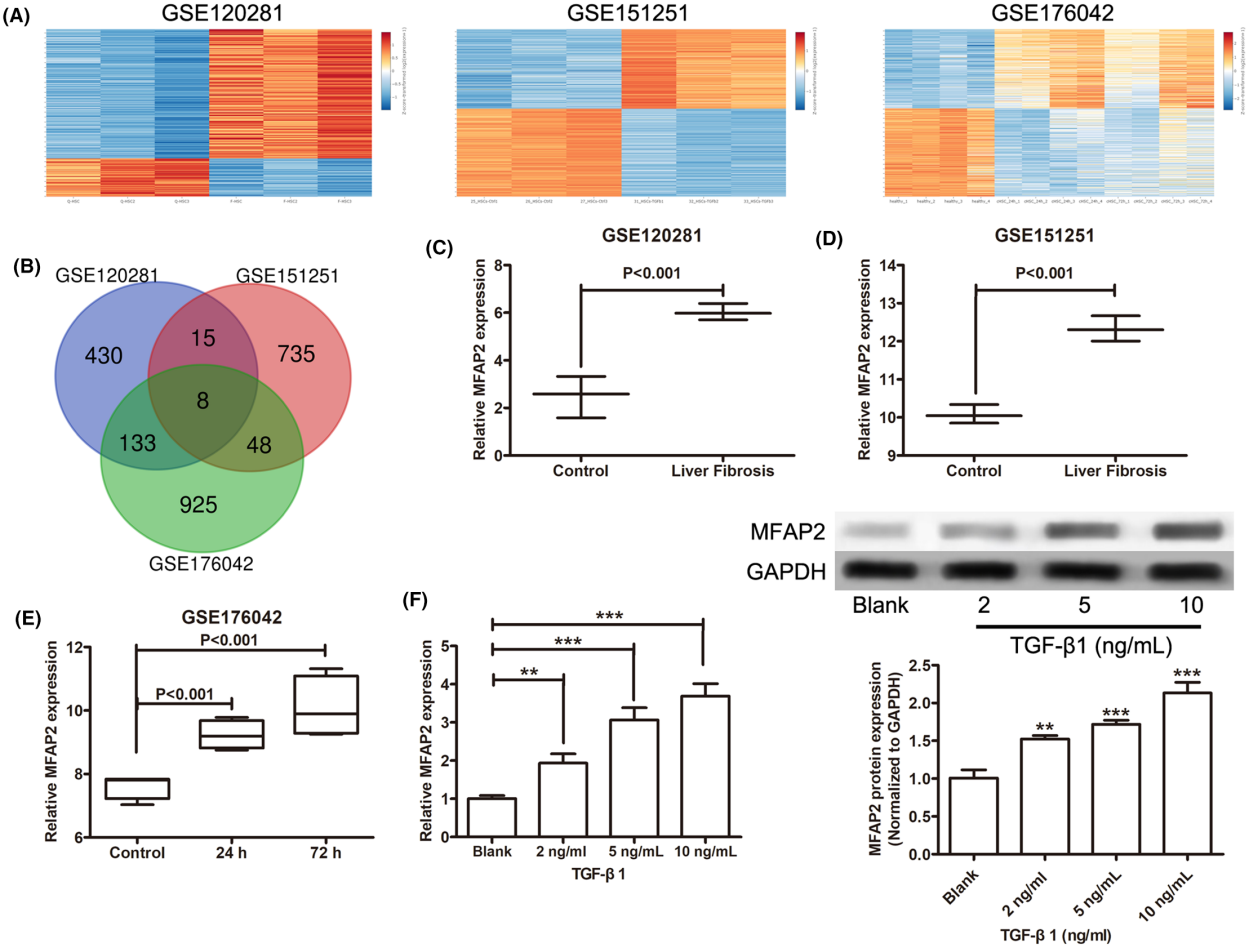
MFAP2 is elevated in CCl_4_‐induced fibrotic liver HSCs and activated HSCs. (A) Heatmaps of differentially expressed genes of GSE120281, GSE151251 and GSE176042 datasheets. (B) Venn diagram of common DEGs in the three datasets. (C–E) The expression of MFAP2 in GSE120281, GSE151251 and GSE176042 datasheets. (F) The mRNA and protein expression levels of MFAP2 in LX‐2 cells with TGF‐β1 treatment. **p < 0.01, ***p < 0.001.

To determine DEGs of HSCs in CCl_4_‐induced liver fibrosis in mouse models, RNA‐sequencing datasheets (GSE120281, GSE151251 and GSE176042) containing quiescent or fibrotic HSCs were analysed with the criterion of |log fold change| >2 and *p* values <0.05 (Figure 1A). Using Venn diagram online tool, 8 upregulated genes (PTGS2, SSC5D, MFAP2, SLIT3, PRG4, SERPINE2, PI16, ACTA2) associated with HSCs activation were identified from the three datasets (Figure 1B). The expression of MFAP2 was significantly increased in CCl_4_‐induced fibrotic mouse liver HSCs compared with normal liver HSCs according to GSE120281 and GSE176042 datasets (Figure 1C,E), as well as human HSCs treated with TGF‐β1 treatment (Figure 1D). Furthermore, MFAP2 expression was significantly upregulated in LX‐2 cells with TGF‐β1 treatment both at mRNA and protein level (Figure 1F), which suggested that abnormal MFAP2 expression might participate in the progression of liver fibrosis.

### 3.2 Knockdown of MFAP2 inhibits HSCs to activation

**FIGURE 2:**
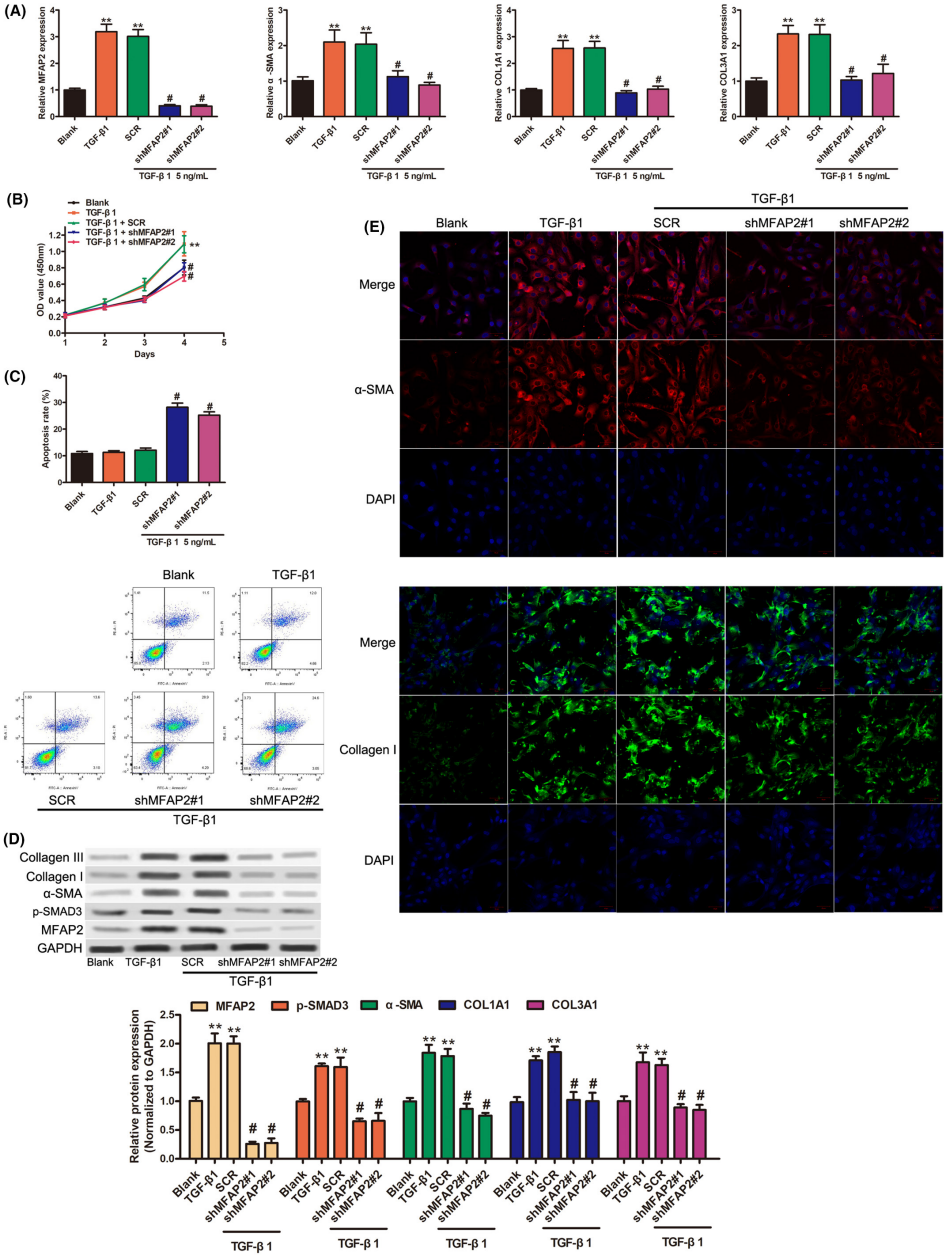
Knockdown of MFAP2 inhibited TGF‐β‐activated HSCs. (A) Relative mRNA levels of MFAP2, α‐SMA, COL1A1 and COL3A1 were determined by RT‐qPCR as indicated treatment. (B) Cell viability and (C) cell apoptosis were determined in LX‐2 cells after 5 ng/mL TGF‐β1 with or without MFAP2 silencing. (D) Protein expression of MFAP2, p‐SMAD3, α‐SMA, COL1A1 and COL3A1 in LX‐2 cells after 5 ng/mL TGF‐β1 with or without MFAP2 silencing. (E) Represent immunofluorescence images of α‐SMA and COL1A1 as indicated treatment. **p < 0.01 versus the blank groups; #*p* < 0.05 versus the SCR group.

To directly evaluate the role of MFAP2 in HSCs activation, endogenous MFAP2 expression in LX‐2 cells were knocked down by lentivirus‐mediated shRNA. As shown in Figure 2A, knockdown of MFAP2 could significantly decrease the mRNA expression of MFAP2, as well as the expression of fibrotic genes such as p‐SMAD3, α‐SMA, COL1A1 and COL3A1. CCK‐8 assay was used in the evaluation of the cell viability, as shown in Figure 2B, there was no significant difference in cell viability after TGF‐β1 treatment. However, there was a significant decrease in cell viability after MFAP2 silencing (Figure 2B). Similarly, cell apoptosis was assessed using flow cytometry and the results showed a significant increase of cell apoptosis rate was observed compared with the blank and TGF‐β1 groups (Figure 2C). However, there was no statistical significance between the blank and TGF‐β1 groups (Figure 2C). Western blot analysis verified the efficiency of MFAP2 knockdown and the protein expression of α‐SMA, COL1A1 and COL3A1 was induced by TGF‐β1 (Figure 2D). Interestingly, the increased of fibrotic genes were restricted when MFAP2 was knocked down (Figure 2D). Immunofluorescent staining also revealed that the increase expression levels of α‐SMA and COL1A1 induced by TGF‐β1 stimulation was partially suppressed by MFAP2 silencing (Figure 2E). These results demonstrated that MFAP2 silencing significantly inhibited HSC activation in vitro.

### 3.3 MFAP2 modulates TGF‐β1‐mediated accumulation of extracellular matrix in LX‐2 cells

**FIGURE 3:**
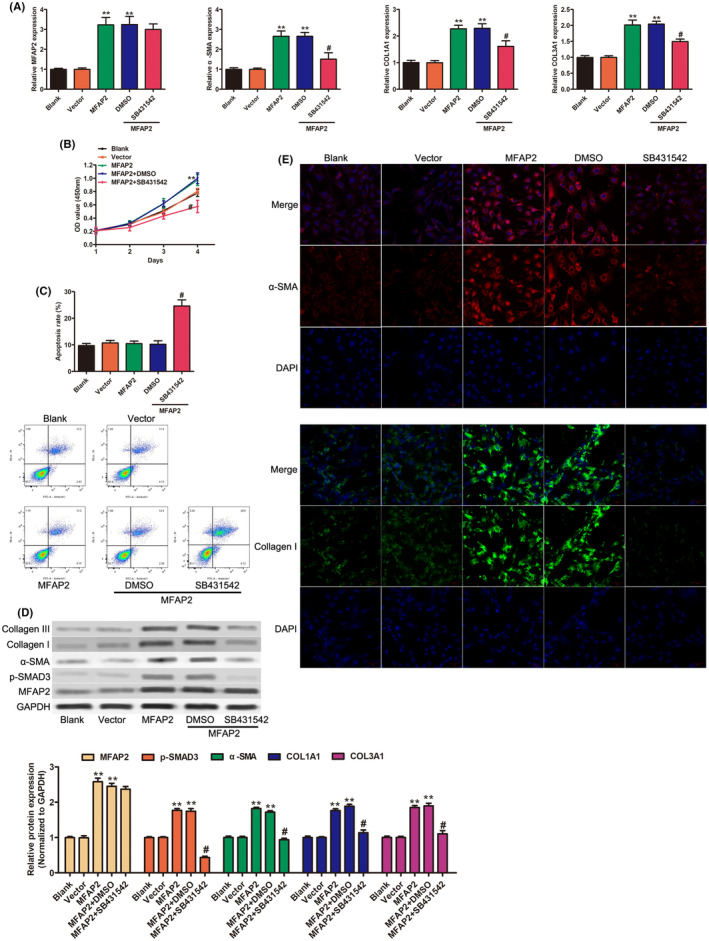
MFAP2 induced activation of HSCs by TGF‐β1/Smad3 pathway. LX‐2 cells were transduced with vector lentiviruses or MFAP2 lentiviruses, then 10 μM SB431542 (TGF‐β1/Smad inhibitor) was added into MFAP2 overexpressed LX‐2 cells. (A) The mRNA level of MFAP2, α‐SMA, COL1A1 and COL3A1 in LX‐2 cells as the indicated treatment. (B) Cell proliferation and (C) Cell apoptosis was assessed by CCK‐8 and flow cytometry assay, respectively. (D) Western blotting of MFAP2, p‐Smad3, α‐SMA, COL1A1 and COL3A1. (E) Expression of α‐SMA and COL1A1 was measured by immunofluorescence. **p < 0.01 versus control vector group, #*p* < 0.05 versus MFAP2 group.

Considering the upregulated MFAP2 expression in fibrotic HSCs, it prompted us to determine whether overexpression of MFAP2 contributed to HSCs activation. RT‐qPCR and western blot exhibited a significant increase level of MFAP2 after MFAP2 overexpression (Figure 3A,D). The mRNA expression level of pro‐fibrotic gene (α‐SMA, COL1A1 and COL3A1) was strongly increased after MFAP2 overexpression (Figure 3A). CCK‐8 assay showed that the overexpression of MFAP2 promoted the proliferation of LX‐2 cells (Figure 3B). However, the apoptotic cells were not remarkably changed using flow cytometry (Figure 3C). Western blot analysis also showed that overexpression of MFAP2 promoted the expression of α‐SMA, COL1A1 and COL3A1 in LX‐2 cells (Figure 3D). Enhanced α‐SMA and COL1A1 expression was observed in MFAP2 overexpressed LX‐2 cells by immunofluorescence (Figure 3E). The canonical TGF‐β/SMAD pathway was reported involved in liver fibrosis.^20^, ^21^ Interestingly, the protein levels of p‐Smad3 was dramatically increased in LX‐2 cells after MFAP2 overexpression (Figure 3D). To further investigate the effect of TGF‐β1/Smad signalling on HSC activation after MFAP2 overexpression, SB431542 (TGF‐β1 inhibitor) was used to explore the role of TGF‐β1/Smad signalling in MFAP2‐indued the activation of HSCs. Notably, pharmacological inhibition of TGF‐β1/Smad signalling could significantly attenuated the upregulation of α‐SMA, COL1A1 and COL3A1 at transcriptional level (Figure 3A). Furthermore, SB431542 effectively inhibited the cell proliferation induced by MFAP2 (Figure 3B), as well as indued the apoptosis level of LX‐2 cells after MFAP2 overexpression (Figure 3C). Moreover, MFAP2‐induced expression of fibrotic markers was partly abolished by SB431542 treatment using western blot (Figure 3D) and immunofluorescence (Figure 3E). These results suggest that MFAP2 promotes upregulation of fibrosis‐related genes by activating the TGF‐β1/Smad3 signalling pathway.

### 3.4 Expression of MFAP2 and its correlation with FBN1

**FIGURE 4:**
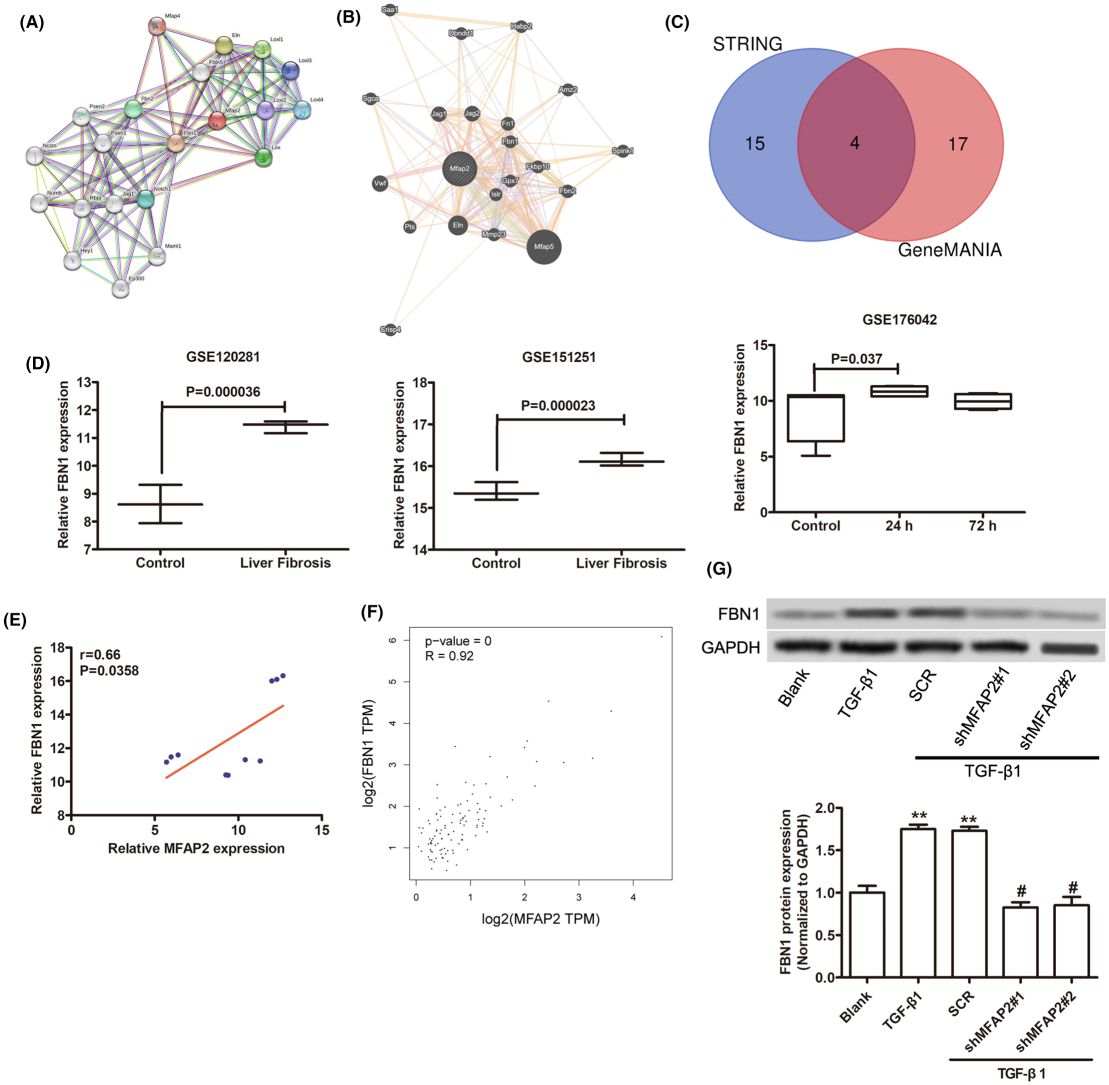
Expression of FBN1 and its correlation with MFAP2. (A) Gene–Gene interaction of MFAP2 predicted by STRING. (B) Gene–Gene Interaction of MFAP2 predicted by GeneMANIA. (C) The Venn diagram of common genes between STRING and GeneMANIA datasheets. (D) The mRNA level of FBN1 in GSE120281, GSE151251 and GSE176042 datasheets. (E, F) Correlation analysis results of MFAP2 with FBN1 in GSE120281, GSE151251 and GSE176042 datasheets (E) and TCGA database (F). (G) The expression of FBN1 in LX‐2 cells after 5 ng/mL TGF‐β1 with or without MFAP2 silencing.

To elucidate the underlying mechanism of MFAP2‐induced activation of LX‐2 cells, STRING and GeneMANIA were used to predict the interaction genes with MFAP2. As shown in Figure 4A, there were 21 potential genes correlated genes with MFAP2, including Eln, Ep300, Fbln5, Fbn1 and Fbn2. Gene–gene interaction analysis showed 20 correlated genes with MFAP2 by GeneMANIA, such as Mfap5, Fbn1, Fbn2 and Fn1 (Figure 4B). There were four common genes were identified using Venn diagram online tool, including Fbn2, Jag1, Eln and Fbn1 (Figure 4C). Then Fbn1 was selected due to the increase expression of FBN1 in GSE120281, GSE151251 and GSE176042 datasheets (Figure 4D). A positive correlation was observed in GSE120281, GSE151251 and GSE176042 datasets (Figure 4E). Consistently, FBN1 positively correlated with the expression of MFAP2 in normal liver tissues based on TCGA datasets (Figure 4F). Furthermore, FBN1 was upregulated after treatment with TGF‐β1 (Figure 4G). However, MFAP2 knockdown partly attenuated the increase of FBN1 induced by TGF‐β1, suggesting that MFAP2 could affect the stability of FBN1 (Figure 4G).

### 3.5 MFAP2 drives the expression of FBN1 via TGF‐β/Smad3 signalling pathway

**FIGURE 5:**
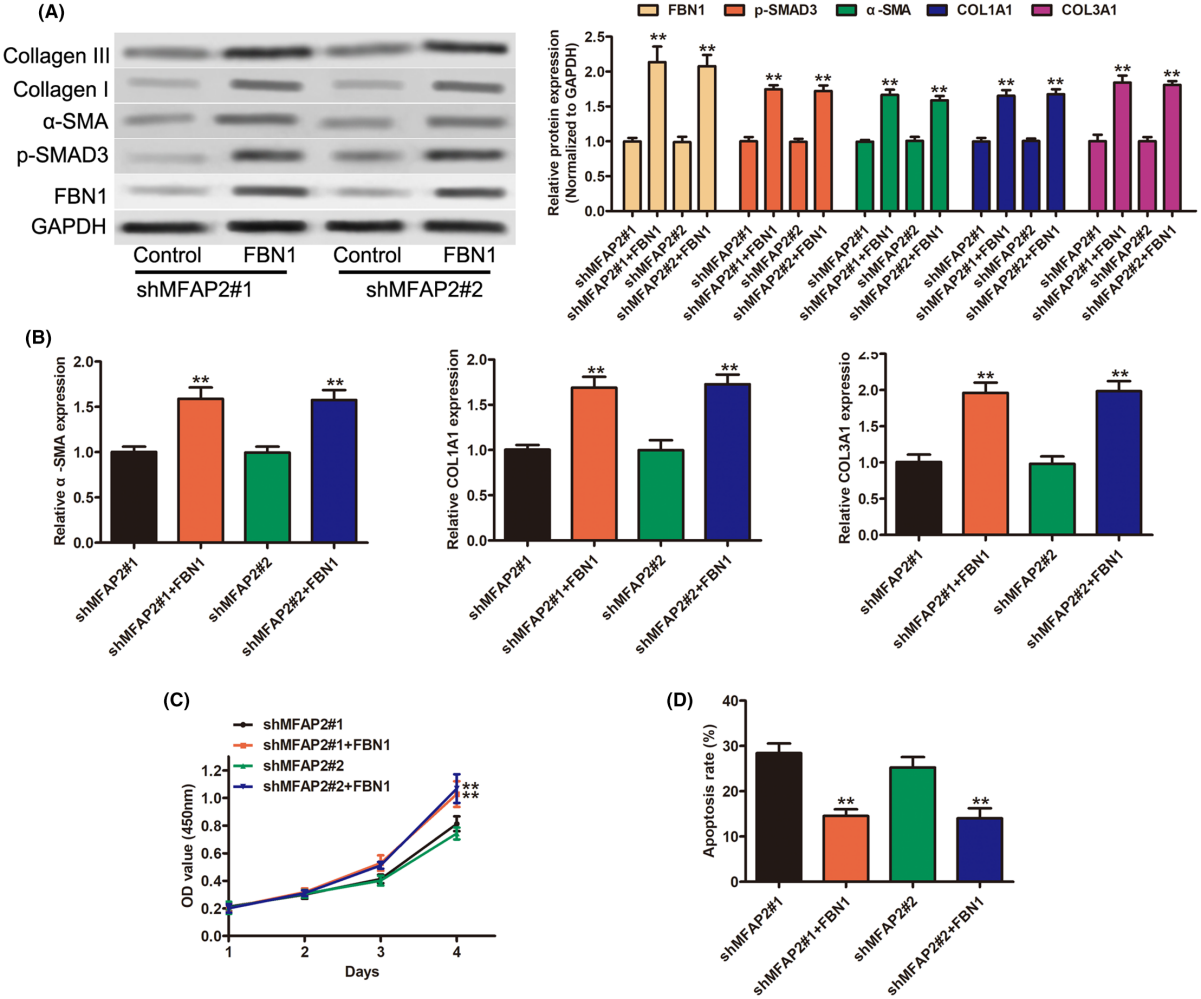
MFAP2 drives HSCs activation through the FBN1/TGF‐β1/SMAD3 signalling pathway. Stable MFAP2 knockdown LX‐2 cells were transfected with control or FBN1. (A) Protein expression of FBN1, p‐Smad3, α‐SMA, COL1A1 and COL3A1 by western blot. (B) RT‐qPCR detection of α‐SMA, COL1A1 and COL3A1 mRNA levels. (C) Cell proliferation (D) cell apoptosis.

Previous reports have shown that FBN1 was remarkably upregulated in several organ fibrosis and related to the development of liver fibrosis.^22^, ^23^, ^24^ Then we explored whether FBN1 was required for the biological functions of MFAP2. FBN1 was overexpressed in MFAP2 stable knockdown LX‐2 cells. As shown in Figure 5A, FBN1 was significantly increased after FBN1 overexpression. Western blotting analysis also showed that FBN1 could partly reverse the decreased expression of fibrotic markers induced by MFAP2 silencing, as well as p‐Smad3 (Figure 5A). Similarly, the mRNA level of fibrotic markers (α‐SMA, COL1A1 and COL3A1) was increased after FBN1 overexpression (Figure 5B). Furthermore, overexpression of FBN1 remarkably restored the cell proliferation (Figure 5C) and inhibited cell apoptosis (Figure 5D) as compared to control, suggesting that MFAP2 mediated TGF‐β/Smad3 signalling pathway and fibrotic markers during liver fibrosis by upregulating the expression of FBN1.

### 3.6 Inhibition of MFAP2 attenuate hepatic injury and fibrosis induced by CCl_4_ in mice

**FIGURE 6:**
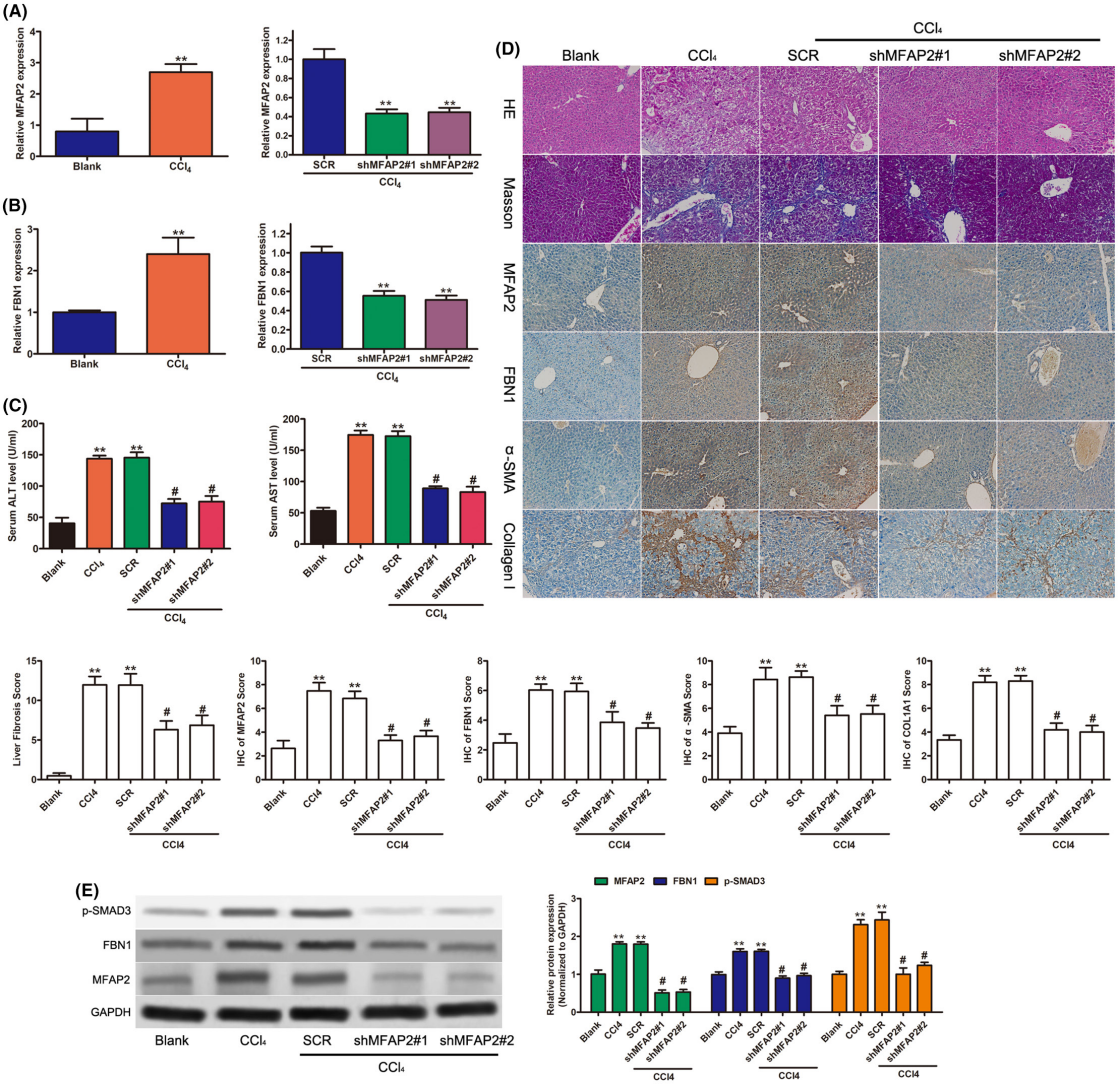
Inhibition of MFAP2 relieved liver pathological damage and improved liver function. Mice (each group *n* = 8) were assigned into five groups: blank, CCl_4_, CCl_4_ with SCR, CCl_4_ with shMFAP2#1 and CCl_4_ with shMFAP2#2. Serum and liver tissues were harvested for following experiments. (A) The mRNA level of MFAP2. (B) The mRNA level of FBN1. (C) The alteration of serological ALT and AST concentration. (D) Liver histological alterations using haematoxylin–eosin and Masson staining and IHC of MFAP2, FBN1, α‐SMA and COL1A1. (E) Protein expression of p‐SMAD3, MFAP2 and FBN1 in different groups of mice. **p < 0.01 versus blank group; #*p* < 0.01, versus CCl_4_ treated group.

To further conform the role of MFAP2 on liver fibrosis, a mouse model of hepatic fibrosis in C57BL/6J mice (*n* = 8) caused by injecting CCl_4_ was established. The mRNA level of MFAP2 and FBN1 were significantly higher in mouse liver fibrotic tissues than in blank mice fed a standard diet (Figure 6A,B). In corroboration with the histological data, the protein levels of MFAP2 were significantly increased, as well as of FBN1 (Figure 6D). The increase of AST and ALT levels were observed in mice treated with CCl_4_ (Figure 6C). Liver injury and fibrosis were assessed by haematoxylin–eosin staining and Masson's trichrome staining. haematoxylin–eosin staining showed the presence of inflammatory cell infiltration, lobular disorder and hepatocytes necrotic in the liver (Figure 6D). Masson's trichrome staining indicated that collagen was increased and deposited (Figure 6D). Similar results were observed for mice treated with CCL_4_ and SCR lentiviruses. However, lentiviruses with targeting MFAP2 showed the decrease mRNA level of MFAP2 and FBN1 (Figure 6A,B). Furthermore, shMFAP2 treatment remarkably suppressed the expression of AST and ALT, which was elevated in the model group (Figure 6C). Moreover, pathological examinations showed that shMFAP2 treatment led to a decrease inflammatory cell infiltration and collagen deposition (Figure 6D). In addition, immunohistochemical staining revealed that FBN1 and fibrotic markers (α‐SMA and COL1A1) were markedly downregulated after MFAP2 silencing (Figure 6D). Western blot also showed the expression of p‐SMAD3, MFAP2 and FBN1 in hepatic tissues (Figure 6E). Our data suggested that MFAP2 might be involved in liver fibrosis by regulating the expression of FBN1.

## 4 DISCUSSION

Liver fibrosis is a common pathological feature of chronic inflammatory liver damage and will develop into liver cirrhosis, liver failure and HCC.^13^, ^25^ Activation of HSCs played critical roles in the development of liver fibrosis due to the secretion of ECM (mainly Type I collagen), which contributed to liver fibrosis.^8^, ^26^ Increasing studies have shown that targeting HSCs would slow down fibrosis regression by decreasing the production and deposition of ECM.^27^, ^28^, ^29^ Nevertheless, the underlying molecular mechanisms and the potential targets for treatment have not well characterized in‐depth.

In this study, three independent GEO databases (between quiescent and fibrotic HSCs by high throughput sequencing) were used to identify eight common upregulated genes. Among the upregulated genes, MFAP2 were obtained for further experiments due to its localization and function.^9^, ^12^ Previous studies have shown that MFAP2 was involved in controlling growth factor signal transduction and interacted with the TGF‐β superfamily members to promote tumour progression, particularly in relation to epithelial‐mesenchymal transition (EMT) and ECM remodelling.^9^, ^30^, ^31^, ^32^ In our present study, MFAP2 was found to be increased in CCl_4_‐induced fibrotic HSCs and TGF‐β1‐activated HSCs. In TGF‐β1‐stimulated LX‐2 cells, knockdown of MFAP2 partially attenuated TGF‐β1‐induced HSCs viability and production of ECM components. Furthermore, overexpression of MFAP2 facilitated proliferation and activation of HSCs, mediated by TGF‐β1/Smad3 signalling. Moreover, Integrative network analysis revealed that the MFAP2 could upregulate the expression of FBN1. FBN1 was reported to involve in ECM organization in liver fibrosis based on bioinformatics analysis.^33^ FBN1 was reported to interact with other ECM proteins to regulate the bioavailability of TGFβ family members to coordinate elasticity to connective tissues.^34^ Enforcing FBN1 expression could partially reversed the effects of MFAP2 silencing on HSCs in vitro. Treatment with lentiviruses with targeting MFAP2 significantly extenuated the pathological changes induced by CCl_4_ and improved the liver function of mice.

Previous studies have shown that MFAP2 bind with fibrillin‐1 and TGFβ contributes to induce the expression of genes linked to cell adhesion, motility, metabolism, gene expression, development and signal transduction.^13^, ^35^ MFAP2 was reported to promote EMT by activating TGF‐β/SMAD2/3 signalling pathway in gastric cancer cells.^14^ In this study, MFAP2 was found to be positively correlated with FBN1, and MFAP2 promoted the expression of FBN1. Furthermore, functional assays showed that overexpression of FBN1 could significantly reverse the inhibition effects in LX‐2 cells after MFAP2 knockdown, which indicated that FBN1 was required for MFAP2‐regulated activation of HSCs. FBN1 was also shown to regulate storage and activation of TGF‐β in the human idiopathic pulmonary fibrosis.^36^ Our in vivo experiments also showed the decrease expression of FBN1 after MFAP2 knockdown.

Our study has its limitations. We only focused on activation of HSCs affected by MFAP2 and the role of MFAP2 in collagen deposits and ECM organization. We did not investigate the effects of MFAP2 on collagen deposits and ECM organization in hepatocytes. In future research, we will explore the effects of MFAP2 on collagen deposits and ECM organization in hepatocytes. Second, targeted deletion of MFAP2 in HSCs and mice lacking MFAP2 in HSCs were needed to verify the roles of MFAP2 in collagen deposits and ECM organization.

## 5 CONCLUSIONS

**FIGURE 7:**
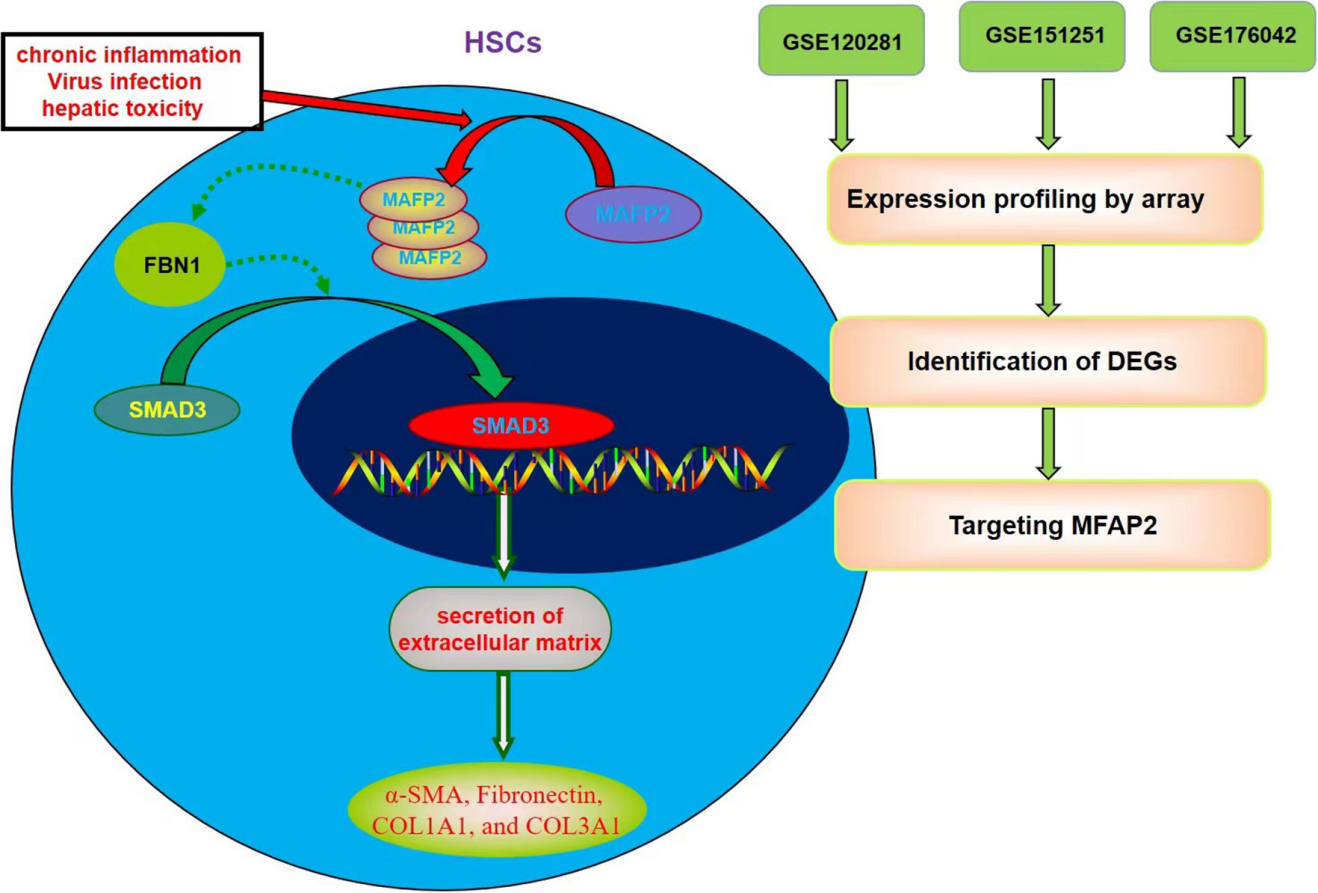
A schematic illustration of the role of MFAP2 in modulating the expression of FBN1 to activate TGF‐β1/SMAD3 in the hepatic stellate cells (HSCs) underlying liver fibrosis.

In conclusion, to the best of our knowledge, this is the first study to demonstrate the potential role of MFAP2 in the activation of HSCs during liver fibrosis. MFAP2 was significantly overexpressed in liver fibrosis and activated HSCs after continuous inflammatory stimulation. MFAP2 bind FBN1 to triggered TGF‐β1/Smad signalling pathway to activate HSCs to product ECM (Figure 7). Our findings suggest that targeting MFAP2 may be a promising interventional strategy for liver fibrosis in the future.

## AUTHOR CONTRIBUTIONS

**Yonghong Sun:** Funding acquisition (equal); investigation (equal); project administration (equal); supervision (equal); validation (equal); writing – review and editing (equal). **Xingxing Chen:** Data curation (equal); validation (equal); writing – original draft (equal). **Lili Chen:** Investigation (equal). **Baixin Bao:** Data curation (equal); investigation (equal). **Chunming Li:** Data curation (equal); formal analysis (equal); methodology (equal); supervision (equal); visualization (equal). **Yongning Zhou:** Conceptualization (lead); resources (lead).

## FUNDING INFORMATION

This research was funded by Science and Technology Project of Gansu Province (grant number 20JR10RA385, 18YF1WA040) and the Science Foundation of Gansu Province People's Hospital (grant no.19SYPYB‐24, 18GSSY3‐4).

## CONFLICT OF INTEREST STATEMENT

None.

## Supporting information


Table S1.

## Data Availability

The datasets generated during the current study are available from the corresponding author upon reasonable request.

## References

[1] Li B , Wang H , Zhang Y , et al. Current perspectives of neuroendocrine regulation in liver fibrosis. Cells. 2022;11(23):3783. 36497043 10.3390/cells11233783 PMC9736734

[2] Kamada Y , Nakamura T , Isobe S , et al. SWOT analysis of noninvasive tests for diagnosing NAFLD with severe fibrosis: an expert review by the JANIT forum. J Gastroenterol. 2022;58:1‐19. 36469127 10.1007/s00535-022-01932-1 PMC9735102

[3] Abd EE , Zakaria AY . Targeting HSP47 and HSP70: promising therapeutic approaches in liver fibrosis management. J Transl Med. 2022;20(1):544. 36435779 10.1186/s12967-022-03759-z PMC9701392

[4] Kisseleva T , Brenner D . Molecular and cellular mechanisms of liver fibrosis and its regression. Nat Rev Gastroenterol Hepatol. 2021;18(3):151‐166. 33128017 10.1038/s41575-020-00372-7

[5] Tacke F , Trautwein C . Mechanisms of liver fibrosis resolution. J Hepatol. 2015;63(4):1038‐1039. 26232376 10.1016/j.jhep.2015.03.039

[6] Nakano Y , Kamiya A , Sumiyoshi H , Tsuruya K , Kagawa T , Inagaki Y . A deactivation factor of fibrogenic hepatic stellate cells induces regression of liver fibrosis in mice. Hepatology. 2020;71(4):1437‐1452. 31549421 10.1002/hep.30965

[7] Trivedi P , Wang S , Friedman SL . The power of plasticity‐metabolic regulation of hepatic stellate cells. Cell Metab. 2021;33(2):242‐257. 33232666 10.1016/j.cmet.2020.10.026 PMC7858232

[8] Tsuchida T , Friedman SL . Mechanisms of hepatic stellate cell activation. Nat Rev Gastroenterol Hepatol. 2017;14(7):397‐411. 28487545 10.1038/nrgastro.2017.38

[9] Craft CS . MAGP1, the extracellular matrix, and metabolism. Adipocyte. 2015;4(1):60‐64. 26167404 10.4161/adip.32209 PMC4496976

[10] Craft CS , Broekelmann TJ , Mecham RP . Microfibril‐associated glycoproteins MAGP‐1 and MAGP‐2 in disease. Matrix Biol. 2018;71‐72:100‐111. 10.1016/j.matbio.2018.03.006 PMC6128786 29524629

[11] Chen E , Larson JD , Ekker SC . Functional analysis of zebrafish microfibril‐associated glycoprotein‐1 (Magp1) in vivo reveals roles for microfibrils in vascular development and function. Blood. 2006;107(11):4364‐4374. 16469878 10.1182/blood-2005-02-0789 PMC1895789

[12] Weinbaum JS , Broekelmann TJ , Pierce RA , et al. Deficiency in microfibril‐associated glycoprotein‐1 leads to complex phenotypes in multiple organ systems. J Biol Chem. 2008;283(37):25533‐25543. 18625713 10.1074/jbc.M709962200 PMC2533084

[13] Craft CS , Pietka TA , Schappe T , et al. The extracellular matrix protein MAGP1 supports thermogenesis and protects against obesity and diabetes through regulation of TGF‐β. Diabetes. 2014;63(6):1920‐1932. 24458361 10.2337/db13-1604 PMC4030109

[14] Wang JK , Wang WJ , Cai HY , et al. MFAP2 promotes epithelial‐mesenchymal transition in gastric cancer cells by activating TGF‐beta/SMAD2/3 signaling pathway. Onco Targets Ther. 2018;11:4001‐4017. 30034240 10.2147/OTT.S160831 PMC6047603

[15] Silveira NJ , Varuzza L , Machado‐Lima A , et al. Searching for molecular markers in head and neck squamous cell carcinomas (HNSCC) by statistical and bioinformatic analysis of larynx‐derived SAGE libraries. BMC Med Genomics. 2008;1:56. 19014460 10.1186/1755-8794-1-56 PMC2629771

[16] Dong SY , Chen H , Lin LZ , et al. MFAP2 is a potential diagnostic and prognostic biomarker that correlates with the progression of papillary thyroid cancer. Cancer Manag Res. 2020;12:12557‐12567. 33324100 10.2147/CMAR.S274986 PMC7732165

[17] Dewidar B , Meyer C , Dooley S , Meindl‐Beinker AN . TGF‐beta in hepatic stellate cell activation and liver Fibrogenesis‐updated 2019. Cells. 2019;8(11):1419. 31718044 10.3390/cells8111419 PMC6912224

[18] Xu F , Liu C , Zhou D , Zhang L . TGF‐beta/SMAD pathway and its regulation in hepatic fibrosis. J Histochem Cytochem. 2016;64(3):157‐167. 26747705 10.1369/0022155415627681 PMC4810800

[19] Xu Z , He B , Jiang Y , et al. Igf2bp2 knockdown improves CCl(4)‐induced liver fibrosis and TGF‐beta‐activated mouse hepatic stellate cells by regulating Tgfbr1. Int Immunopharmacol. 2022;110:108987. 35820364 10.1016/j.intimp.2022.108987

[20] Schnabl B , Kweon YO , Frederick JP , Wang XF , Rippe RA , Brenner DA . The role of Smad3 in mediating mouse hepatic stellate cell activation. Hepatology. 2001;34(1):89‐100. 11431738 10.1053/jhep.2001.25349

[21] Li Y , Fan W , Link F , Wang S , Dooley S . Transforming growth factor beta latency: a mechanism of cytokine storage and signalling regulation in liver homeostasis and disease. JHEP Rep. 2022;4(2):100397. 35059619 10.1016/j.jhepr.2021.100397 PMC8760520

[22] Lou Y , Tian GY , Song Y , et al. Characterization of transcriptional modules related to fibrosing‐NAFLD progression. SCI Rep‐UK. 2017;7(1):4748. 10.1038/s41598-017-05044-2 PMC5500537 28684781

[23] Fusco C , Nardella G , Augello B , et al. Pro‐fibrotic phenotype in a patient with segmental stiff skin syndrome via TGF‐beta signaling overactivation. Int J Mol Sci. 2020;21(14):5141. 32698527 10.3390/ijms21145141 PMC7404389

[24] Li L , Liao J , Yuan Q , et al. Fibrillin‐1‐enriched microenvironment drives endothelial injury and vascular rarefaction in chronic kidney disease. Sci Adv. 2021;7(5):eabc7170. 33571112 10.1126/sciadv.abc7170 PMC7840119

[25] Popov Y , Schuppan D . Targeting liver fibrosis: strategies for development and validation of antifibrotic therapies. Hepatology. 2009;50(4):1294‐1306. 19711424 10.1002/hep.23123

[26] Hernandez‐Gea V , Friedman SL . Pathogenesis of liver fibrosis. Annu Rev Pathol. 2011;6:425‐456. 21073339 10.1146/annurev-pathol-011110-130246

[27] Fondevila MF , Fernandez U , Heras V , et al. Inhibition of carnitine palmitoyltransferase 1A in hepatic stellate cells protects against fibrosis. J Hepatol. 2022;77(1):15‐28. 35167910 10.1016/j.jhep.2022.02.003

[28] Fu Y , Zhou Y , Mu Y , et al. Testicular orphan receptor 4 induced hepatic stellate cells activation via the regulation of TGF‐beta receptor I/Smad2/3 signaling pathway. Ann Hepatol. 2022;28(1):100775. 36280014 10.1016/j.aohep.2022.100775

[29] Zhang H , Zhou P , Xing W , et al. GLIS2 prevents hepatic fibrosis by competitively binding HDAC3 to inhibit hepatic stellate cell activation. Cell Mol Gastroenterol Hepatol. 2022;15(2):355‐372. 36397300 10.1016/j.jcmgh.2022.10.015 PMC9792572

[30] Zhu S , Ye L , Bennett S , Xu H , He D , Xu J . Molecular structure and function of microfibrillar‐associated proteins in skeletal and metabolic disorders and cancers. J Cell Physiol. 2021;236(1):41‐48. 32572962 10.1002/jcp.29893

[31] Gomez DSI , Ahechu P , Gomez‐Ambrosi J , et al. Decreased levels of microfibril‐associated glycoprotein (MAGP)‐1 in patients with colon cancer and obesity are associated with changes in extracellular matrix remodelling. Int J Mol Sci. 2021;22(16):8485. 34445187 10.3390/ijms22168485 PMC8395192

[32] Broekelmann TJ , Bodmer NK , Mecham RP . Identification of the growth factor‐binding sequence in the extracellular matrix protein MAGP‐1. J Biol Chem. 2020;295(9):2687‐2697. 31988245 10.1074/jbc.RA119.010540 PMC7049978

[33] Yang X , Cheng QN , Wu JF , Ai WB , Ma L . Analysis of key genes and related transcription factors in liver fibrosis based on bioinformatic technology. Int J Clin Exp Pathol. 2021;14(4):444‐454. 33936366 PMC8085816

[34] Summers KM , Bush SJ , Davis MR , Hume DA , Keshvari S , West JA . Fibrillin‐1 and asprosin, novel players in metabolic syndrome. Mol Genet Metab. 2023;138(1):106979. 36630758 10.1016/j.ymgme.2022.106979

[35] Walji TA , Turecamo SE , DeMarsilis AJ , Sakai LY , Mecham RP , Craft CS . Characterization of metabolic health in mouse models of fibrillin‐1 perturbation. Matrix Biol. 2016;55:63‐76. 26902431 10.1016/j.matbio.2016.02.006 PMC4992667

[36] Lepparanta O , Sens C , Salmenkivi K , et al. Regulation of TGF‐beta storage and activation in the human idiopathic pulmonary fibrosis lung. Cell Tissue res. 2012;348(3):491‐503. 22434388 10.1007/s00441-012-1385-9

