# Supplementary material for: MFAP2 promotes HSCs activation through FBN1/TGF‐β/Smad3 pathway
Source: J Cell Mol Med. 2023 Aug 27;27(21):3235–46. doi: 10.1111/jcmm.17884 (PMC10623529; doi:10.1111/jcmm.17884)
Supplement: Supplementary file 1 — Table S1. [file JCMM-27-3235-s001.docx]

| **Table S1 Primer sequence for the study** | |  |
| --- | --- | --- |
| Gene | Forward Sequence (5′-3′) | Reverse Sequence (5′-3′) |
| GAPDH | TGACCTCAACTACATGGTCTACA | CTTCCCATTCTCGGCCTTG |
| MFAP2 | CTGGAGACTGAGCCTACCGA | GGCTTGTGGATGGAGTAGAGG |
| α-SMA | ACCCAGCACCATGAAGATCA | TCTGCTGGAAGGTAGACAGC |
| Collagen Ⅰ | TCCCTGGAATGAAGGGACAC | CTCTCCCTTAGGACCAGCAG |
| Collagen Ⅲ | CTGTAACATGGAAACTGGGGAAA | CCATAGCTGAACTGAAAACCACC |
| FBN1 | CTGGAGACTGAGCCTACCGA | GGCTTGTGGATGGAGTAGAGG |
| human |  |  |
| GAPDH | GACGAACATGGGGGCATCAG | GCGTCACCACCATGGAG |
| MFAP2 | GCCGTGAGGAACAGTACCC | TAGAAGCAGACCTCGTTGAGA |
| α-SMA | AAAAGACAGCTACGTGGGTGA | GCCATGTTCTATCGGGTACTTC |
| Collagen Ⅰ | CGCTGGTTTCGATTCAGCT | ACATTGGCATCATCAGCCCG |
| Collagen Ⅲ | GGTAGCCCTGGTGAGAGAGG | CGGAGCCCCTCTTTCTCCTT |
| FBN1 | GCGGAAATCAGTGTATTGTCCC | CAGTGTTGTATGGATCTGGAGC |
| SCR | CCGGCGCCAAGCCCTTGTCTTCACACTCGAGTGTGAAGACAAGGGCTTGGCGTTTTTG | AATTCAAAAACGCCAAGCCCTTGTCTCACAACTCGAGTGTGAAGACAAGGGCTTGGCG |
| sh-MFAP2#1 | CCGGGCCTTGCAAACAGTGTCTCAACTCGAGTTGAGACACTGTTTGCAAGGCTTTTTG | AATTCAAAAATTGAGACACTGTTTGCAAGGCCTCGAGGCCTTGCAAACAGTGTCTCAA |
| sh-MFAP2#2 | CCGGGACAACCCAGACTACTATGATCTCGAGATCATAGTAGTCTGGGTTGTCTTTTTG | AATTCAAAAAATCATAGTAGTCTGGGTTGTCCTCGAGGACAACCCAGACTACTATGAT |
| pcDNA-3.1-FBN1 | AAAGGATCCATGCGTCGAGGGCGTCTG | AAAGCTAGCTTATTCATGCTGCATCCGC |
| pCDH-MFAP2 | AAAGGATCCATGAGAGCTGCCTACCTCTTC | AAAGCTAGCCTAGCAGCTCCCACAGCTCC |
